# Deciphering the Effect of Hyaluronic Acid/Collagen Hydrogel for Pain Relief and Tissue Hydration in a Rat Model of Intervertebral Disc Degeneration

**DOI:** 10.3390/polym16182574

**Published:** 2024-09-11

**Authors:** Rusydi Mohd Razak, Nur Arina Amira Harizal, Mohammad Ali Zuhdi Azman, Najwa Syakirah Mohd Redzuan, Raed H. Ogaili, Ahmad Hafiz Kamarrudin, Muhammad Fakhrullah Mohamad Azmi, Nur Aqilah Kamaruddin, Aminatul Saadiah Abdul Jamil, Sabarul Afian Mokhtar, Isma Liza Mohd Isa

**Affiliations:** 1 Department of Anatomy, Faculty of Medicine, Universiti Kebangsaan Malaysia, Cheras, Kuala Lumpur 56000, Malaysia; a174157@siswa.ukm.edu.my (R.M.R.); a173739@siswa.ukm.edu.my (N.A.A.H.); a175041@siswa.ukm.edu.my (M.A.Z.A.); a173573@siswa.ukm.edu.my (N.S.M.R.);; 2 Department of Orthopaedics and Traumatology, Faculty of Medicine, Universiti Kebangsaan Malaysia, Cheras, Kuala Lumpur 56000, Malaysiadrsam@ppukm.ukm.edu.my (S.A.M.); 3 Health Industry Technology Programme, Faculty of Science and Technology, Universiti Sains Islam Malaysia, Nilai 71800, Malaysia; muhd.fakhrullah05@gmail.com (M.F.M.A.); aminatul.abduljamil@usim.edu.my (A.S.A.J.); 4 CÚRAM, SFI Research Centre for Medical Devices, School of Medicine, University of Galway, H91 W2TY Galway, Ireland

**Keywords:** low back pain, degenerative disc disease, hydrogel, disc repair

## Abstract

Intervertebral disc (IVD) degeneration is one of the primary causes of low back pain, causing disability; hence, there is no regenerative nature of the current treatments. Hyaluronic acid (HA) was reported to facilitate tissue repair and alleviate pain. Herein, we determined the therapeutic effect of HA and type II collagen (COLII) hydrogel for tissue repair targeting pain in IVD degeneration. We implanted HA/COLII hydrogel following surgically induced disc injury at coccygeal levels in the rat tail model of pain. We assessed the efficacy of the HA/COLII hydrogel in reducing pain behaviour by using the von Frey assessment, protein expression of growth-associated protein (GAP) 43 for sensory nerve innervation, and disc hydration by magnetic resonance imaging (MRI). We observed the anti-nociceptive effect of the HA/COLII hydrogel in alleviating mechanical allodynia in rats. There was an inhibition of sensory hyperinnervation indicated by the GAP43 protein in the treatment group. We revealed an increase in T1ρ mapping of MRI, indicating that the hydrogel restored disc hydration in vivo. Our findings suggest the HA/COLII hydrogel alleviates pain behaviour, inhibits hyperinnervation and promotes disc hydration for tissue repair, implying that it is a potential candidate for the treatment of degenerative disc-associated low back pain.

## 1. Introduction

Low back pain (LBP) is the leading cause of musculoskeletal disability, putting a significant strain on worldwide healthcare systems. According to the World Health Organization, low back pain affects about 570 million individuals worldwide, and it accounts for 7.4% of all healthy years lost to disability worldwide (YLD) [1]. Discogenic low back pain affects between 26% and 42% of patients with chronic back pain; hence, intervertebral disc (IVD) degeneration is one of the most common causes [2]. IVDs comprise the nucleus pulposus (NP), annulus fibrosus (AF) and cartilaginous endplates. The NP has a gel-like texture and is positioned in the centre of the intervertebral disc, composed of around 66% to 86% water, with the remaining fraction made up of collagen (mainly type II) and proteoglycans. It contributes to the strength and flexibility of the spine. Meanwhile, the AF is the outer lamellae ring that surrounds the NP and can withstand compressive forces from the NP. Type I collagen, proteoglycans, glycoproteins and elastic fibres make up the majority of it [3].

**Figure 1:**
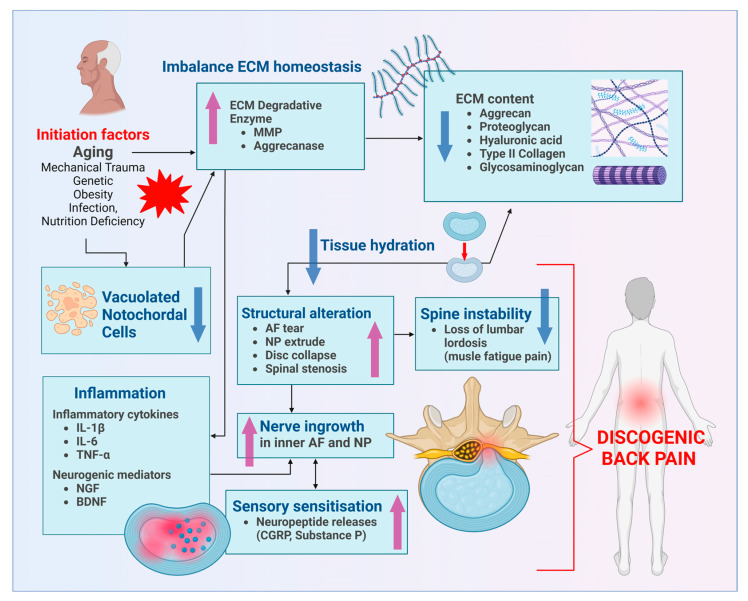
An illustration of the pathophysiology of IVD degeneration underlying discogenic back pain. The blue arrow pointing down indicates a decrease, while the purple arrow pointing up indicates an increase.

The pathological mechanism behind IVD degeneration involves the overlay of three stages. Stage I (early degeneration) involves an imbalance in the production of ECM in the NP, such as type II collagen and proteoglycan, with increased levels of enzymes (matrix metalloproteinases, MMP and aggrecanases) causing ECM degradation. The ECM degradation will cause disc dehydration and eventually cause disc collapse. MRI was a suitable method to measure disc hydration [4]. Stage II (mild degeneration) is associated with increased production of inflammatory markers such as IL-1β, IL-6, IL-7, IL-2, IL-8, IL-10, IL-4, tumour necrosis factor (TNF)-α and interferon-γ. Stage III (severe degeneration) is associated with the AF tear, causing nerve ingrowth, vascularisation and NP extrusion [5]. Pro-inflammatory cytokines and neurotrophic factors are the inducers of sensory nerve ingrowth into the aneural IVD [6]. IL-1β will induce the expression of nerve growth factor (NGF) and expression of brain-derived neurotrophic factor (BDNF). Nociceptive sensory nerve fibres contain neuropeptides classified as small-diameter NGF-sensitive neurons derived from the dorsal root ganglia (DRG) associated with inflammation-induced hyperalgesia [2]. Unmyelinated sensory nerve fibres expressed growth-associated protein 43 (GAP43) [7]. Nerve fibres were sensitised with inflammatory and neurogenic mediators, and the pain signal was transmitted to the peripheral afferent neurons, causing discogenic pain [2,6,7] (Figure 1).

The current treatment options available for IVD degeneration are conservative and surgical, including anti-inflammatories, analgesics, physical therapy, epidural injections, surgical decompression, disc replacement and spinal fusion. However, conservative treatment could not halt the progression of IVD degeneration, whereas surgical treatment carries the risk of surgical complications, postoperative symptom recurrence and adjacent segment degeneration [8]. The pharmacological treatments available for patients with discogenic low back pain are nonsteroidal anti-inflammatory drugs, analgesics and muscle relaxants. While they are beneficial for short-term symptom alleviation [9], long-term use raises the risk of peptic ulcer disease, acute renal damage, stroke and myocardial infarction [10].

Preclinical models of discogenic pain offer a platform for evaluating the efficacy of the proposed treatment. Surgically induced disc injury is now widely used for inducing anatomical changes as the damage to the AF and NP is also known to affect human discs. This injury type is associated with relatively fast degeneration and can be properly controlled. The rat tail model has also been widely used to research disc degeneration because it prevents injury to the adjacent tissue and renders intervention possible [11]. The disc-punctured method was used to cause anatomical distribution of the IVD, which mimicked the pathological features of disc degeneration and measurable pain behavioural development using the von Frey test [7].

Herein, we proposed the biomaterial approach of using an extracellular matrix (ECM)-based hydrogel system to target pain while promoting tissue repair in the IVD. Hydrogels are classified into two types, which are synthetic and natural hydrogels. Natural hydrogels have high cell affinity due to their particular cell action sites, but they are challenging to acquire in large numbers, and their structure and properties are complex to modify. Synthetic hydrogels may be mass-produced and tailored to specific purposes by enhancing their structure and mechanical characteristics throughout manufacturing. However, they lack cell recognition signals and have little cell affinity [8]. In IVD degeneration, a high molecular weight of hyaluronic acid (HA) has been proven to support long-term functional gains by reducing inflammation and pain in various clinical diseases. HA was tested in various pathological conditions, such as ulcerative colitis and liver metastasis [12]. It was also used as a viscosupplementation with intra-articular knee injection in patients with osteoarthritis, which was proven to relieve pain [13]. Our previous study showed that HA hydrogel alleviates pain behaviour, inhibits hyperinnervation, attenuates inflammation and promotes disc hydration in the IVD degeneration model [7]. Collagen has the ability to self-assemble to form fibre, which can form mechanical strength and provide structural support for tissue [14]. Type II collagen polyethylene glycol (PEG)-HA microgels cause adipose-derived stem cells to differentiate toward an NP-like phenotype [15].

In this study, we employed a type II collagen (COLII) hydrogel enriched with HA to target pain and promote tissue hydration for tissue repair. We hypothesised that implantation of the HA/COLII-based hydrogel would alleviate the pain behaviour of mechanical allodynia, promote disc hydration and inhibit sensory hyperinnervation in a surgically induced disc injury in the rat tail of the pain model.

## 2. Materials and Methods

**Figure 2:**
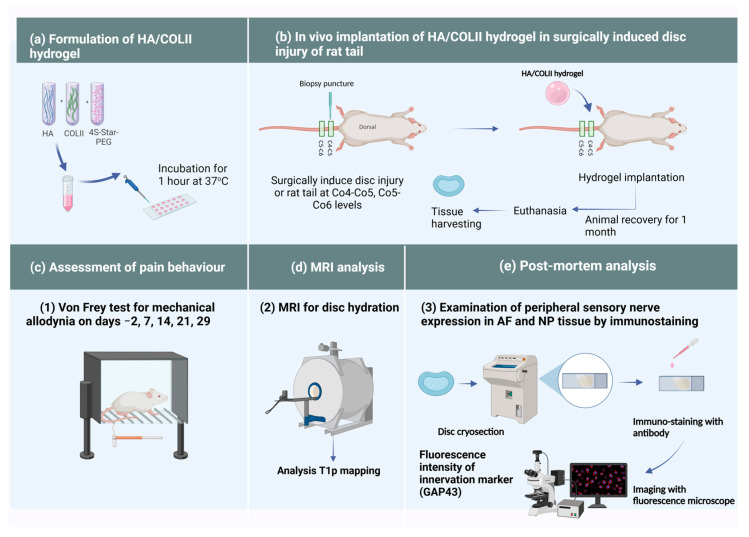
Schematic representation of the experimental plan. (**a**) Formulation of HA/COLII hydrogel. (**b**) In vivo implantation of HA/COLII hydrogel in surgically induced disc injury of rat tail model of pain. (**c**) Von Frey test for pain behaviour assessment. (**d**) MRI for disc hydration. (**e**) Immuno-staining for GAP43 protein.

The methodology of this study is illustrated in Figure 2.

### 2.1. Animals

A total of 18 adult female Sprague Dawley rats were used in this study. The rats were divided into three groups, which were sham control without any surgery (sham), surgically induced injury at coccyx levels Co4-Co5 and Co5-Co6 without HA/COLII-based hydrogel implantation (non-treated injury group) and surgically induced injury [Co4-Co5 + Co5-Co6] with 4 µL of HA/COLII-based hydrogel (2 mg/mL) implantation (treatment group). The rats were grouped randomly with *n* = 6 in each of the three groups. Food and tap water were available ad libitum. Wood shavings were changed frequently during the weekdays to ensure clean and comfortable bedding for them.

### 2.2. Chemicals and Reagents

The sodium hyaluronate (molecular weight, 1.19 × 10^6^ Da) was purchased from Lifecore Biomedical (Chaska, MN, USA). 4S-StarPEG, molecular weight 10,000, was purchased from JenKem Technology Inc. (Plano, TX, USA). Type II collagen (COLII) solution was purchased from Reprocell Inc. (Yokohama, Japan). Teflon tape and Superfrost Plus slides were purchased from Thermo Fisher Scientific (Selangor, Malaysia). The von Frey filaments were purchased from North Coast Medical Inc (Morgan Hill, CA, USA). Rabbit polyclonal anti-GAP43 (ab12274) and Goat Anti-Rabbit IgG H&L (TRITC) (ab6718) were purchased from Abcam (Cambridge, UK). All the other chemicals used were of analytical grade.

### 2.3. Synthesis of HA/COLII-Based Hydrogel

The collagen hydrogel was synthesised as in our previous protocol [16] using a solution of bovine articular-derived type II collagen (COLII) at a concentration of 2 mg/mL. This solution was mixed with a high molecular weight of sodium hyaluronate (HA) at 10 mg/mL. The HA and COLII were mixed at weight ratios of 1:9. For chemical crosslinking, 4S-StarPEG with a molecular weight of 10,000 was added to the mixture at a 1:1 molar ratio. The resulting solution was then micro-dispensed as 4 μL droplets onto a hydrophobic glass slide to form spherical 3D hydrogels. These were incubated at 37 °C for 1 h to complete the crosslinking reaction (Figure 2a). The hydrogels were well characterised for swelling capacity, stability, degradability and cytotoxicity [16] before being used for in vivo implantation in this study.

### 2.4. Mechanical Allodynia by von Frey Test

Rats were placed in the test lab 20 min before starting the test to reduce locomotor activity and stress-induced analgesia during the test. The investigator was blinded to the experimental groups. The von Frey test was performed 2 days pre-operatively for baseline and on days 7, 14, 21 and 29 post-operatively. Each rat was placed in a perspex chamber enclosure for 20 min to minimise their exploratory activity. The 2 g filament was placed at the base of the ventral surface of the rat tail for a maximum of 6 s. The positive response was indicated when the rat responded by flinching, licking, withdrawing or shaking the base of the tail immediately or before the 6 s period ended. A negative response was noted if there was no response. This process was repeated five times with the 2 g filament. If a positive response was obtained with the 2 g filament, the test was repeated with progressively lower-weight filaments until no response is obtained in 5 attempts. The test was continued with increasing filament numbers until there were five positive responses in five attempts for two consecutive filaments. Withdrawal responses were calculated using the formula below [5].

$$\mathrm{Percentage}\;\mathrm{withdrawal}\;\mathrm{response}\;\left(\%\right)=\left(\frac{\mathrm{Number}\;\mathrm{of}\;\mathrm{positive}\;\mathrm{responses}\;}{\mathrm{Number}\;\mathrm{of}\;\mathrm{applications}}\right)\times 100$$

Then, the 50% withdrawal threshold was calculated. Using GraphPad Prism, a histogram graph of von Frey filament weight (g) eliciting a 50% withdrawal threshold versus time (days) was plotted.

### 2.5. Implantation of HA/COLII-Based Hydrogel Following Surgically Induced Disc Injury of Rat Tail Model of Pain

On the surgery day, the rats were given a single subcutaneous dose of the nonsteroidal anti-inflammatory drug carprofen (5 mg/kg) 15 min prior to surgery to manage postoperative pain during the early recovery phase. They were anaesthetised using ketamine (100 mg/kg)/xylazine (10 mg/kg). The effectiveness of anaesthesia was determined by pinching the rats’ toes (loss of pedal withdrawal reflex). The surgical site of the dorsal surface of the rat tail was washed with Betadine solution using gauze. The intervertebral discs Co4-Co5 and Co5-Co6 were identified by palpating the vertebral bodies through the tail skin. The discs between the first two vertebral bodies starting from the base of the tail are the Co4-Co5 and, subsequently, Co5-Co6. A rubber band was applied at the base of the tail to reduce blood flow to the tail. A longitudinal incision was performed on the area identified earlier. The tendons were pushed aside to reveal the AF tissue underneath. After reaching the AF tissue, a 1 mm biopsy puncture was used to induce a punch-in injury to the NP measuring 1 mm in diameter with a depth of 2 m. The injured site was either left alone or implanted with HA/COLII-based hydrogel. The wound was sutured. The rubber band was removed for blood circulation. All surgical instruments were handled using an aseptic technique.

Post-operatively, the rats were closely monitored until they recovered from the anaesthetic effect. Each rat was placed individually in a cage until their wound healed sufficiently, which took around one week. Then, three of them in the same group were placed together in a cage for recovery. Wounds were examined carefully to look for signs of inflammation or infection, such as erythema, swelling, or any discharge from the surgical site. Their general health assessment, including body weight, was recorded on days −2, 2, 7, 14 and 28. Animals were kept recovered until 29 days and euthanised for post-mortem analysis.

### 2.6. Magnetic Resonance Imaging (MRI) for Disc Hydration

On day 29 post-operation, the rats were anaesthetised using ketamine (100 mg/kg)/xylazine (10 mg/kg) before an MRI was performed on the rats’ spines (*n* = 3) in each group using a 0.25 T system MRI (Vet MR Grande, Esaote, Florence, Italy). The T1ρ measurement was performed at the selected slice by three-dimensional spoiled gradient recalled echo sequencing using the following parameters: echo time/repetition time (TE/TR) = 4.3/8.7 ms; time of spinlock (TSL) = 0, 20, 40, 60 and 80 ms; spin-lock frequency = 500 Hz; resolution = 0.27 × 0.27 × 3 mm^3^; and acquisition time = 13 min. For T1ρ quantification, regions of interest representing the NP and AF were placed on sagittal images, and T1ρ relaxation times and calculated maps were generated using MATLAB software version R2018a (Mathworks, Natick, MA, USA) with a monoexponential fitting.

### 2.7. Immunostaining of GAP43 Protein for Sensory Innervation

After tissue harvesting, the discs were fixed in 4% paraformaldehyde for 48 h. Samples were decalcified in Kristensen’s solution containing 18% (*v*/*v*) formic acid and 3.5% (*w*/*v*) sodium formate for two weeks at a temperature of 4 °C. Samples were washed under running tap water before being infiltrated with 30% (*w*/*v*) sucrose solution until they sank at 4 °C. Samples were embedded in an optimal cutting temperature (OCT) medium and then snap-frozen in an isopentane bath with liquid nitrogen. The samples were kept at −80 °C before proceeding with a cryostat. They were cryosectioned at 10 µm thick using a HM525 NX cryostat (Thermo Scientific, Runcorn, UK). The tissue sections were collected on Superfrost Plus slides and stored at −80 °C until use. The cryosection slides were warmed from −20 °C to room temperature. A circle surrounding the tissue sections was drawn using a hydrophobic Dako pen. The sections were incubated with proteinase K for 15 min at 37 °C before being washed in PBS-T (PBS with 0.05% Tween 20) three times for five minutes each time. Then, the sections were incubated with 2% BSA for 30 min at room temperature and washed in PBS-T three times for five minutes each time. The sections were then incubated overnight with anti-GAP43 primary antibody (1:100 dilution in PBS-T) and then washed in PBS-T three times for five minutes each time. Subsequently, the sections were incubated for 2 h with Goat Anti-Rabbit IgG H&L (TRITC) (1:200 dilution in PBS-T) before being washed in PBS-T three times for five minutes each time. Then, the sections were counterstained with DAPI (1:1000 in PBS) for five minutes and washed with PBS-T three times for five minutes each time. Sections were mounted with ProLong^®^ gold antifade. A coverslip was applied on top of them. Tissue sections were cured in the dark at 4 °C until fluorescence imaging.

### 2.8. Fluorescence Intensity Quantification

Immuno-stained tissue sections were imaged using a fluorescence microscope OLYMPUS BX53FL (Evident Corporation, Tokyo, Japan), focusing on the area of AF and NP with at least three microscopic views of each slide with three technical and three biological replicates. Fluorescence intensity quantification was performed using ImageJ software version 1.48 [National Institutes of Health (NIH)]. The fluorescence image of GAP43 was adjusted to the optimal threshold of positive staining. Fluorescence intensity quantification was performed in the area of interest. The mean value and standard deviation of the fluorescence intensity were plotted.

### 2.9. Statistical Analysis

The statistical differences between each group were analysed using GraphPad Prism version 9.5.1. The von Frey data were analysed by repeated measures ANOVA. The expression of GAP43 protein and T1ρ values were analysed by one-way ANOVA. All ANOVAs were further evaluated with Bonferroni post-hoc analysis, and * *p* < 0.05 was deemed statistically significant. Data are all mean, and error bars indicate SEM.

## 3. Results

### 3.1. HA/COLII-Based Hydrogel Alleviated Mechanical Allodynia

**Figure 3:**
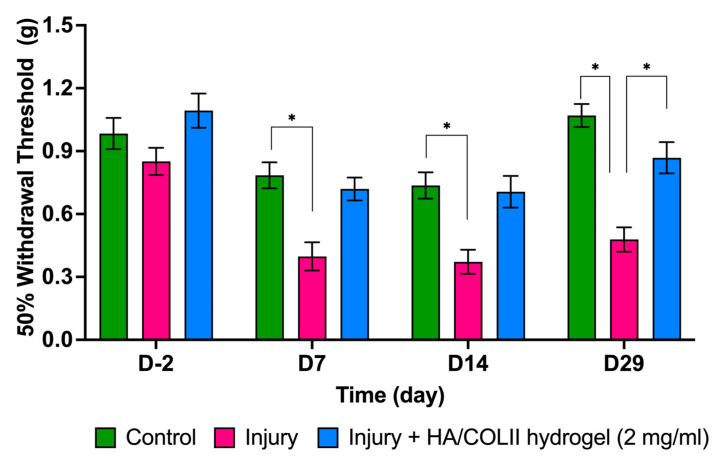
The von Frey assessment revealed that 50% withdrawal was significantly lower in the untreated injury group up to 29 days, but it was higher in the HA/COLII hydrogel-treated injury group. Data are means ± SEM, *n* = 6. * *p* < 0.05 constitutes a significant difference between groups, determined by repeated measure ANOVA and Bonferroni’s post hoc test.

Quantitative sensory testing was conducted after surgical-puncture-induced IVD injury in the rat tail for pain phenotyping. The von Frey test was performed for mechanical allodynia to observe the therapeutic efficacy of the HA/COLII-based hydrogel in alleviating nociceptive behaviour. The von Frey filaments were applied to the ventral base of the tail. There is no significant difference in the 50% withdrawal threshold at the baseline between the three experimental groups of control, untreated injury and hydrogel-treated injury. However, we observed a significantly lower threshold in the untreated injury group compared to the control group up to 29 days. An increased trend of higher thresholds was observed in the hydrogel-treated injury group in comparison to the non-treated injury group, indicating that hydrogel alleviates mechanical allodynia (Figure 3).

### 3.2. Inhibition of Hyperinnervation Post-Implantation of HA/COLII Hydrogel

**Figure 4:**
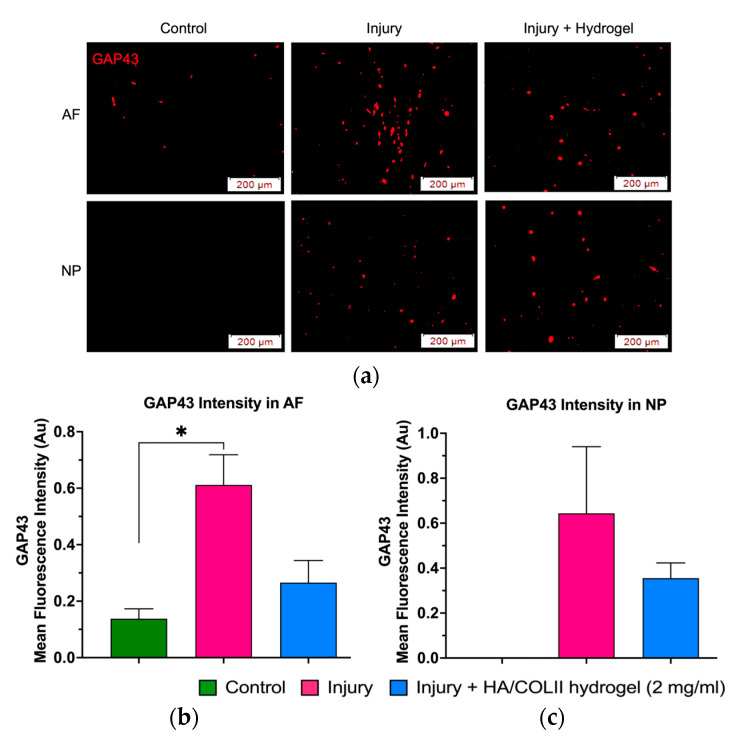
The GAP43 staining in the AF and NP tissues. (**a**) Fluorescent microscopy demonstrated the red stain of the expression of GAP43 protein in the AF and NP tissue for sensory nerve ingrowth. (**b**) The mean fluorescence intensity of GAP43 was lower in the hydrogel-treated injury group compared to the untreated injury group in the AF (**b**) and NP (**c**). Data are means ± SEM, *n* = 3. * *p* < 0.05 constitutes a significant difference between groups, determined by one-way ANOVA and Bonferroni’s post hoc test.

We observed the expression of GAP43 protein in the AF compared to the NP tissue. The mean fluorescent intensity of GAP43 protein was higher in the non-treated injury group of the AF and NP tissue compared to the control uninjured group of the AF and NP, which was lower. In contrast, the GAP43 mean fluorescent intensity was lower in the hydrogel-treated injury group compared to the non-treated injury group in the AF and NP. This finding suggests the inhibitory effect of the HA/COLII hydrogel on injury-induced peripheral sensory hyperinnervation (Figure 4).

### 3.3. HA/COLII Hydrogel Promoted Disc Hydration

**Figure 5:**
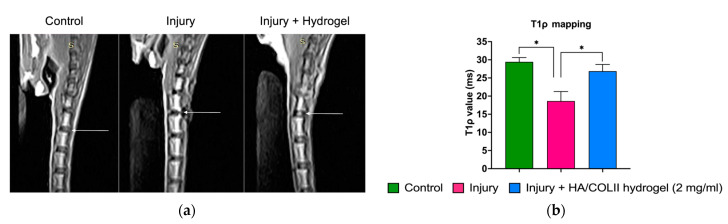
The MRI analysis for disc hydration. (**a**) A representation of MRI images on T1ρ mapping. The arrow indicates the intervertebral discs of control, injury and treatment, respectively. (**b**) The histogram shows that the T1ρ value of the hydrogel-treated injury group was higher compared to the non-treated injury group. Data are means ± SEM, *n* = 3. * *p* < 0.05 constitutes a significant difference between groups, determined by one-way ANOVA and Bonferroni’s post hoc test.

We employed MRI to assess the hydration status in the IVD. The T1ρ mapping of the MRI was higher in the control, which reflects the hydrophilicity of the ECM composition in the IVD. However, it was decreased in the untreated injury group. We observed a higher T1ρ value in rats treated with hydrogel, suggesting that hydrogel restores disc hydration (Figure 5).

## 4. Discussions

The von Frey test applied various filaments, generating a distinct and constant force, indicating mechanical allodynia in the injured rat. We revealed a persistent pain behaviour of mechanical allodynia in rats following disc injury. The lowered threshold of von Frey’s assessment suggests a chronic pain phenotype caused by intervertebral disc degeneration and associated discogenic pain. This finding is consistent with our previously established model of pain induced by disc injury in the rat tail model, which showed increased thermal hyperalgesia and mechanical allodynia in rats [7].

We demonstrated alleviation of mechanical allodynia in rats following the implantation of HA/COLII hydrogel over a month. The hydrogel was enriched with a high-molecular-weight HA molecule previously shown to exert an analgesic effect. This observation was consistent with a previous study that demonstrated that an HA solution reduces pain behaviour in a mouse model by targeting the nociceptive channel of Transient receptor potential vanilloid 1 (TRPV1) [17].

Following tissue injury, pro-inflammatory mediators such as IL-1ß and TNF-α are released, which induce the expression of NGF to induce nerve ingrowth, and that sensitises nociceptors, initiating nociceptive signalling to the spinal cord, which causes pain [6]. Previous studies have shown the ingrowth of small, unmyelinated sensory nerve fibres into the inner third of the AF and the NP with chronic low back pain. Importantly, these fibres have been shown to express GAP43 and substance P [18]. Consistent with our finding, we observed an increased expression of GAP43 in the disc injury group, indicating injury-induced hyperinnervation. Using this model, we observed an inhibitory effect of HA/COLII hydrogel towards hyperinnervation. HA is a non-sulphated glycosaminoglycan, and a previous study had shown that chondroitin sulfate proteoglycan inhibited neurite outgrowth [19]. Previously, it has been demonstrated that aggrecan binds to HA and forms large aggregates, inhibiting neuronal growth in the lumbar IVD [20].

The initiation of IVD degeneration started when there was an extensive reduction of vacuolated notochordal cells in the NP [2]. During IVD maturation, the larger notochordal cells undergo morphology changes and functional shifts to smaller fibrochondrocyte-like cells. The reduction of notochordal cells during ageing contributes to matrix changes and decreases the cellularity of the NP [2,21]. This leads to the alteration and degradation of the ECM content. Using MRI, we analysed the T1ρ mapping, which reflects the proteoglycan deposition in the IVD. Proteoglycan molecules have a high affinity for water due to their negative charges, which attract and bind water molecules, mainly to maintain disc hydration. Herein, we observed a decrease of increase in disc hydration following disc injury. The hydrogel-treated group showed increased hydration, suggesting the HA/COLII hydrogel promotes tissue repair. This is consistent with the previous study, which demonstrated that HA hydrogel increases the expression of type II collagen and aggrecan in the NP [22].

## 5. Conclusions

Our findings indicate that HA/COLII-based hydrogel alleviates mechanical allodynia, inhibits injury-induced sensory hyperinnervation and promotes disc hydration for tissue repair. This may imply a potential therapeutic approach for intervertebral disc degeneration in managing low back pain.

## Data Availability

The data presented in this study are available on request from the corresponding author. The data are not publicly available due to the patent process.
